# Randomized trial of electronic health record implemented AI risk prediction in kidney transplant care

**DOI:** 10.1038/s41746-026-02757-5

**Published:** 2026-05-14

**Authors:** Bilgin Osmanodja, Jakob Joachim Spencker, Ömer Ege Ömeroğlu, Zeineb Sassi, Roland Roller, Sascha Vu-Eickmann, Hannah Thomas, Aljoscha Burchardt, Michael Hahn, Tabea Ott, Peter Dabrock, Sebastian Möller, Klemens Budde, Anne Herrmann

**Affiliations:** 1 Department of Nephrology and Medical Intensive Care, Charité–Universitätsmedizin Berlin, Berlin, Germany; 2 Department of Epidemiology and Preventive Medicine, University of Regensburg, Regensburg, Germany; 3 St Vincent’s Hospital, Sydney, University of New South Wales, Sydney, NSW Australia; 4 German Research Center for Artificial Intelligence, Berlin, Germany; 5 Institute of General Practice, University Hospital Regensburg, Regensburg, Germany; 6 Chair of Systematic Theology II (Ethics), Friedrich-Alexander-Universität Erlangen-Nürnberg, Erlangen, Germany; 7 Department of Systematic Theology and the Study of Religions, University of Vienna, Vienna, Austria; 8 Department of Ethics and Law in Medicine, University of Vienna, Vienna, Austria; 9 Quality and Usability Lab, Technical University of Berlin, Berlin, Germany; 10 Department of Internal Medicine III, University Hospital Regensburg, Regensburg, Germany; 11 Bavarian Cancer Research Center, Regensburg, Germany

**Keywords:** Diseases, Health care, Medical research, Nephrology

## Abstract

Artificial intelligence (AI)-based risk prediction is increasingly implemented in clinical care, but randomized evidence on communication and shared decision-making (SDM) outcomes is limited. In the single-center PRIMA-AI trial, 76 kidney transplant recipients with estimated glomerular filtration rate <30 mL/min/1.73 m² were randomized 1:1 to usual care or usual care plus an electronic health record (EHR)-integrated machine-learning model predicting 1-year graft loss risk. The primary outcome was patient-reported conversations about treatment options after graft loss during 12 months. Conversation frequency did not differ between groups (intervention 14/36 [39%] vs control 16/40 [40%]; chi-square *p* = 1.00). No significant between-group differences were observed for secondary clinical, SDM-related, relationship, or distress outcomes. Post-study user feedback suggested low and variable tool uptake with workflow barriers. Passive EHR availability of AI risk estimates did not improve communication or SDM-related outcomes. Future interventions should strengthen workflow integration and directly support SDM. Trial Registration: ClinicalTrials.gov number, NCT0605651, registered 2023-09-21.

## Introduction

Artificial intelligence (AI)–based risk prediction models are increasingly embedded into electronic health records (EHRs) to support clinical decision-making. Yet evidence that such models improve care processes and patient-centered outcomes remains limited. In particular, predictive performance does not necessarily translate into changed clinical behavior, improved communication, or better shared decision-making (SDM) in routine practice^1,2^.

Kidney transplantation is the preferred therapy for patients with chronic kidney disease (CKD) and kidney failure. Yet eventually 57% of kidney transplant recipients (KTR) experience graft loss, meaning failure of the kidney transplant, while the remainder dies with a functioning graft^3–5^. In patients with CKD experiencing kidney failure (before transplantation), choosing the appropriate kidney replacement therapy is an important preference-sensitive decision and implementing SDM has been shown to leave more patients satisfied with their choice^6–9^.

As graft function declines, KTR face similar preference-sensitive decisions, including (re)initiating hemodialysis or peritoneal dialysis, pursuing retransplantation, or opting for palliative care^10,11^. For KTR approaching graft loss, however, structured and evidence-based SDM interventions are unavailable. In this situation, timely discussions about treatment options after graft loss are recommended but appear to occur infrequently in routine post-transplant care, with prior reports describing rates of 13%^12^. A recent KDIGO (Kidney Disease Improving Global Outcomes) guideline recommends shared decision-making and advance care planning during allograft decline, but evidence on effective implementation strategies remains sparse^11^.

Machine-learning models for graft loss prediction have shown promising accuracy and could provide an actionable signal for clinicians when short- to mid-term risk increases^13–16^. If integrated into the EHR, individualized risk estimates may function as a low-threshold digital nudge to initiate timely conversations about prognosis, options, and preferences. Comparable approaches have been shown to be efficacious in oncology by triggering serious illness conversations and hereby reducing end-of-life systemic therapy^17,18^. Early consultation about patient preferences regarding kidney replacement therapy may not only improve patient satisfaction, but also reduce emergency admissions and central venous catheter placement for dialysis initiation, which has increased risk of infectious complications^7–9^. Whether this type of AI-enabled decision support changes communication frequency or SDM-related outcomes in post-kidney transplant care is unknown.

PRIMA-AI (Prospectively Investigating the Impact of AI on Shared Decision-Making in Post-Kidney Transplant Care) is a randomized trial evaluating whether EHR-delivered AI graft-loss risk estimates influence communication and SDM-related outcomes in patients with impaired graft function^19^. This article reports randomized trial results, including the primary outcome.

## Results

**Fig. 1:**
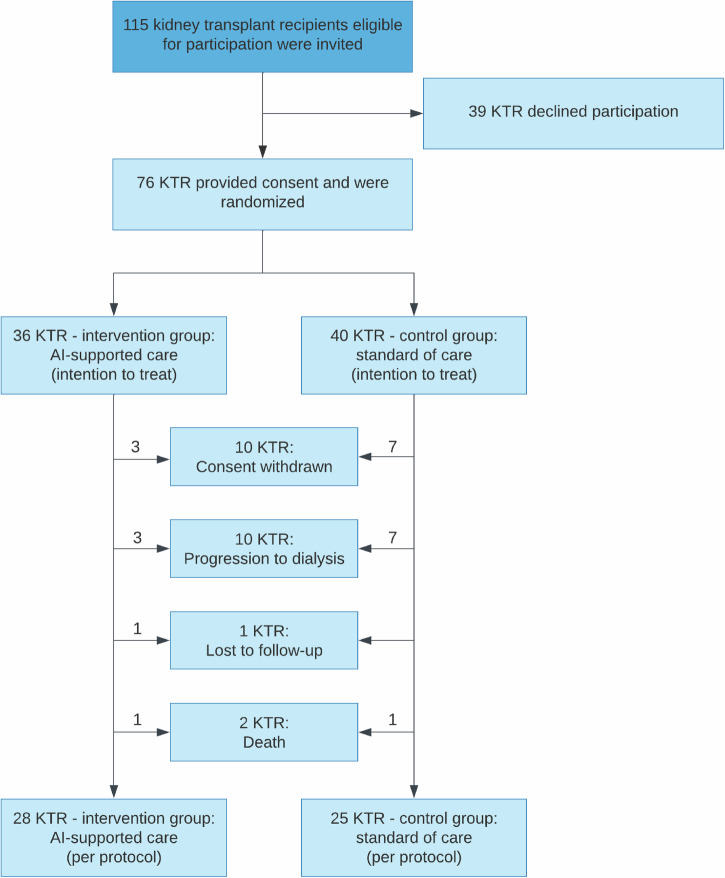
CONSORT flow chart. The Flowchart summarizes the number of patients invited for participation, randomized patients and reasons for dropout over the 12-months study course. KTR, kidney transplant recipient.

From 325 patients prescreened, 115 patients were invited for participation, from which 76 patients were included and randomized between January 24, 2024, and September 18, 2024. The cohort was predominantly born in Germany, German-speaking and of German nationality (born in Germany: 70/76, 92%; German mother tongue: 71/76, 93%; German nationality: 74/76, 97%). Resembling most transplant cohorts, 24/76 participants (32%) were female. Regarding education and occupation, 71% (54/76) reported having finished high school education or higher, while 92% (70/76) reported having any job qualification, from which 42% (32/76) reported having a university degree or comparable job qualification. Patients in the control and intervention group had similar characteristics (Table 1). During the follow-up period of 12 months, 2 patients died and 10 patients experienced graft loss. Additionally, 10 patients withdrew consent for further study participation and 1 patient was lost to follow-up (Fig. 1).

**Table 1:** Baseline characteristics of the study cohort as mean ± standard deviation or relative (absolute) frequency in %

Baseline demographics		Intervention (*n* = 36)	Control (*n* = 40)
Patient age in years		56.4 ± 10.4	57.7 ± 13
Sex	male	66.7% (24)	70% (28)
	female	33.3% (12)	30% (12)
eGFR (ml/min/1.73m^2^)		27.1 ± 9.7	24.6 ± 8
Nationality/Origin	German nationality	100% (36)	95% (38)
	Born in Germany	91.7% (33)	92.5% (37)
	Mother tongue German	94.4% (34)	92.5% (37)
	both parents born in Germany	83.3% (30)	85% (34)
	no answer	-	2.5% (1)
Personal status	married	63.9% (23)	65% (26)
	civil partnership	11.1% (4)	10% (4)
	unmarried	19.4% (7)	12.5% (5)
	widowed/divorced	5.6% (2)	10% (4)
	no answer	-	2.5% (1)
Highest school degree	none	8.3% (3)	7.5% (3)
	lower secondary school diploma	13.9% (5)	20% (8)
	high school diploma	30.6% (11)	17.5% (7)
	(Fach-)abitur (IB)	44.4% (16)	50% (20)
	other/no answer	2.8% (1)	5% (2)
Highest job qualification	job training completed	58.3% (21)	42.5% (17)
	university degree/master’s certificate	38.9% (14)	45% (18)
	none	2.8% (1)	7.5% (3)
	no answer	-	5% (2)
Netto household income	< 500 €	2.8% (1)	5% (2)
	500-999€	13.9% (5)	2.5% (1)
	1000–1499 €	8.3% (3)	17.5% (7)
	1500–1999 €	2.8% (1)	12.5% (5)
	2000–2499 €	13.9% (5)	12.5% (5)
	2500–2999 €	13.9% (5)	2.5% (1)
	3000–3499 €	11.1% (4)	10% (4)
	≥ 3500 €	33.3% (12)	27.5% (11)
	no answer	-	10% (4)

eGFR, estimated glomerular filtration rate; IB, International Baccalaureate.

### Primary outcome

The primary outcome was the proportion of patients who self-reported having a conversation about the treatment options after graft loss with their treating transplant physician. In the intention-to-treat (ITT) analysis, 16/40 (40%) in the control group compared to 14/36 (39%) in the intervention group recalled such a conversation over the study period of 12 months (**χ**²(1, *N* = 76) = 0, *p* = 1.00). In the per-protocol analysis, 11/25 (44%) in the control group compared to 13/28 (46%) in the intervention group recalled such a conversation (**χ**²(1, *N* = 53) = 0, *p* = 1.00). There were no differences in the primary outcome between the two groups, irrespective of the analysis chosen.

### Medical outcomes

In total, 10 patients developed graft loss, 7/40 (17.5%) in the control group and 3/36 (8.3%) in the intervention group (*p* = 0.32, Fisher’s exact test). Of these, four (40%) required in-hospital emergency dialysis (Control: 2/7 (28.6%), Intervention: 2/3 (66.7%), *p* = 0.5). The dialysis access was an arteriovenous fistula in 5/10 (50%) patients, a permanent central venous catheter in 2/10 (20%) patients, a temporary central venous catheter in 1/10 (10%) patient, and a peritoneal dialysis catheter in 2/10 (20%) patients, without significant differences between intervention and control group (Table 2).

**Table 2:** Between group differences in the primary outcome and prespecified secondary clinical outcomes

Outcomes	No/total No. (%) of patients		OR	*P* value
	Control	Intervention		
Primary outcome				
Conversation about treatment options after graft loss	16/40 (40%)	14/36 (39%)	0.96 (0.34–2.64)	1.00
Secondary outcomes–medical				
Graft loss	7/40 (17.5%)	3/36 (8.3%)	0.43 (0.07–2.10)	0.32
Emergency dialysis	2/7 (28.6%)	2/3 (66.7%)	4.17 (0.14–351)	0.50
Dialysis initiation via arteriovenous fistula	5/7 (71.4%)	0/3 (0%)	0	0.17
Dialysis initiation via permanent central venous catheter	0/7 (0%)	2/3 (66.7%)	n.a.	0.07
Dialysis initiation via temporary central venous catheter	1/7 (14.3%)	0/3 (0%)	0	1.00
Dialysis initiation via peritoneal catheter	1/7 (14.3%)	1/3 (33.3%)	2.65 (0.03–273)	1.00

OR, odds ratio. Dialysis-related outcomes were assessed within the patients who returned to dialysis.

### Control preferences scale

**Fig. 2:**
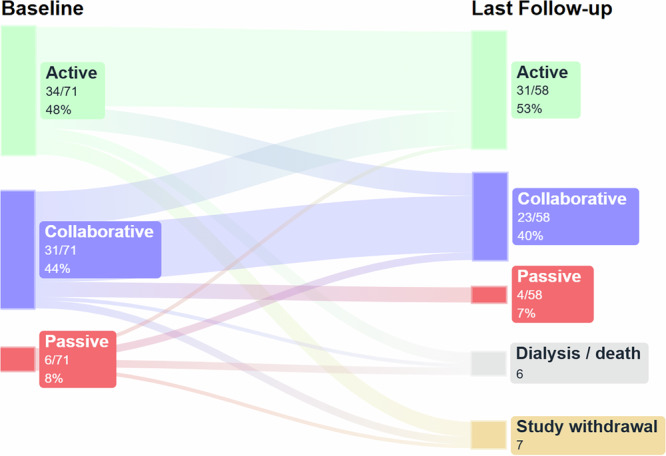
Sankey diagram of decisional preferences according to control preferences scale. The diagram shows decisional preferences (active, collaborative or passive decisional roles) of kidney transplant recipients with impaired graft function according to the control preferences scale over time for the entire study cohort, irrespective of study intervention and potential confounders.

From 76 patients enrolled, 71 (93%) had valid baseline questionnaires for the control preferences scale (CPS). At baseline, decisional role preferences clustered in the active and collaborative categories (34/71 [48%] and 31/71 [44%], respectively), whereas passive preferences were uncommon (6/71 [8%]). From 71 patients with baseline CPS, 6 patients had no further valid questionnaire due to medical events (4 required dialysis, 2 patients died), and 7 patients withdrew consent for further study participation after the baseline visit. Of the 53 patients who completed the study per protocol, plus 5 additional patients who provided at least one follow-up CPS measurement before dropout, 58 patients had valid CPS data for analysis. From those, we analyzed the most recent follow-up visit to track changes in patient preference throughout time. From these, 31/58 (53%) patients preferred an active role in medical decision making, 23/58 (40%) preferred a collaborative role, while 4/58 (7%) preferred a passive role (Fig. 2).

Within the follow-up visits, we found no significant effect of the study intervention or visit number on decision making preference (CPS) (*n* = 157 visits in *N* = 61 patients, *p* = 0.70 and *p* = 0.54, respectively), actual decisional experiences (CPS-post) (*n* = 129 visits in *N* = 54 patients, *p* = 0.78 and *p* = 0.15, respectively) or the divergence between preferred and actual decision-making (CPS–CPS-post) (*n* = 129 visits in *N* = 54 patients, *p* = 0.68 and *p* = 0.53, respectively).

Including all study visits for which both the CPS and CPS-post were answered, the intraclass correlation between actual and preferred roles with respect to the specific decision shown in Table 3 was moderate (*n* = 154 visits in *N* = 63 patients, *r* = 0.45, *p* < 0.001). When preferred and experienced decision roles were compared on the 5-point CPS scale, patients’ experienced role was, on average, more clinician-led than their preferred role (mean preferred-experienced difference: −0.23, SD 0.69).

**Table 3:** Absolute and relative frequencies of decision-making preferences among kidney transplant recipients over time using the control preferences scale (3-point and 5-point)

Preferred Role	Item Description, *n* (%)	Baseline (*n* = 71)	Follow-up (*n* = 58)
Active	I prefer to make the final selection about which renal replacement therapy I will receive	6 (8.5%)	9 (15.5%)
	I prefer to make the final selection of my renal replacement therapy after seriously considering my doctor’s opinion	28 (39.4%)	22 (37.9%)
Collaborative	I prefer that my doctor and I share responsibility for deciding which renal replacement therapy is best for me	31 (44.6%)	23 (39.7%)
Passive	I prefer that my doctor makes the final decision about which renal replacement therapy will be used, but seriously considers my opinion	6 (8.5%)	4 (6.9%)
	I prefer to leave all decisions regarding renal replacement therapy to my doctor	0 (0%)	0 (0%)

### CollaboRATE score

**Fig. 3:**
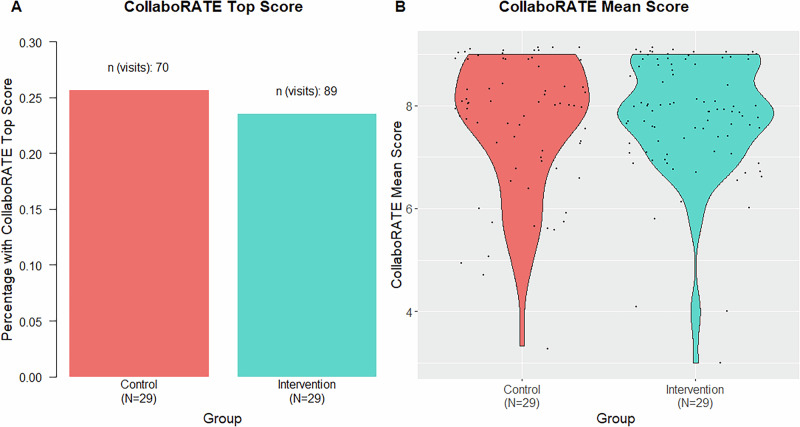
CollaboRATE top score and mean scores grouped by study intervention. The diagram shows CollaboRATE scores grouped by intervention excluding baseline visit with **A** CollaboRATE Top score calculated as the number of visits where all 3 questions were rated with a maximum score of 9, and **B** CollaboRATE mean score calculated as the mean of all visits after baseline per patient.

The CollaboRATE mean score at baseline was 7.83 based on 71 valid measurements, while the CollaboRATE top score was 28.1%. There were no significant differences in CollaboRATE mean and top scores between physicians with at least 10 valid measurements. The study intervention had no effect on CollaboRATE mean and top scores, independent of the covariates chosen in the linear mixed-effects (LME) model (Fig. 3).

**Fig. 4:**
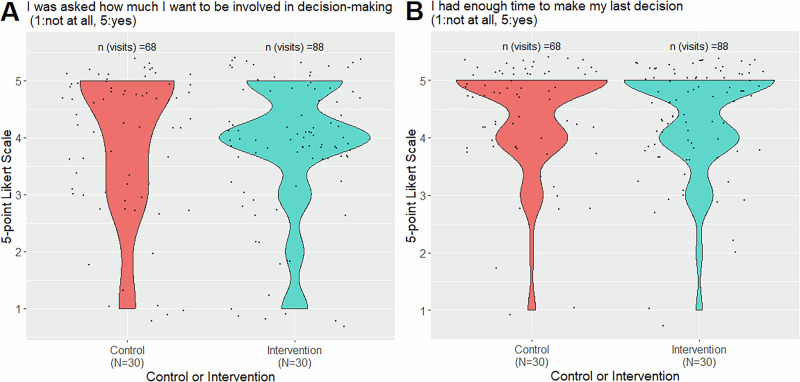
Additional survey items regarding decision-making grouped by study intervention. Agreement with the following two sentences rated on a 5-point Likert scale with 1 - “no, not at all” to 5 - “yes, absolutely” regarding the last decision and for all visits excluding the baseline visit **A** “I was asked how much I want to be involved in decision-making”, **B** “I had enough time to make my last decision”.

In two separate items, patients reported if they were asked how much they want to participate in decision-making and if they were given sufficient time to do so, both regarding the last decision made (Fig. 4). The majority of patients reported to have been asked about their preferred enrollment with no differences between groups (overall mean, intervention: 3.84 SD 1.16, control: 4.02, SD 1.25) and have been given enough time for the decision (overall mean, intervention: 4.33 SD 0.92, control: 4.38, SD 0.95). The study intervention had no effect on both metrics.

### Patient doctor relationship (PDRQ-9)

**Fig. 5:**
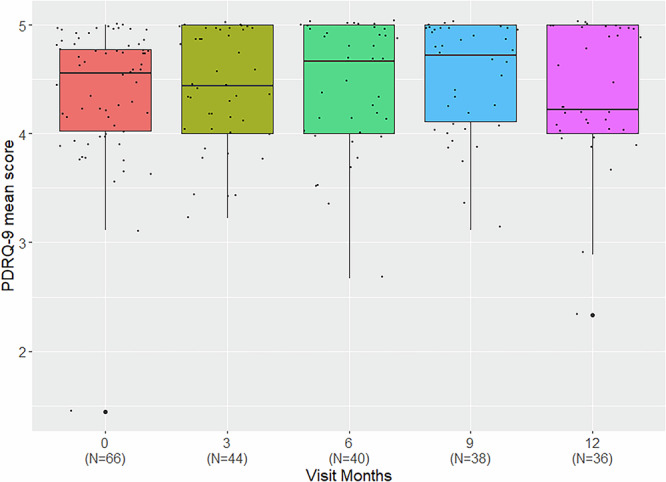
Patient-doctor relationship questionnaire (PDRQ-9). Boxplots showing PDRQ-9 mean score of all items over time.

Mean PDRQ-9 scores were high for all visits (median [interquartile range; IQR]–Baseline: 4.56 [0.78], M3: 4.44 [1], M6: 4.67 [0.94], M9: 4.67 [0.94], M12: 4.22 [1]) as shown in Fig. 5. In a LME model treating patients and physicians as random effects, neither visit number, nor treatment group (for visits month 3–month 12) showed significant effect on mean PDRQ-9 scores or on any single item of the PDRQ-9.

### Self-reported general health and distress thermometer (DT)

At baseline, 7.9% (6/76) of the patients assessed their general health as bad, 42.1% (32/76) as mediocre, 39.5% (30/76) as good, and 1.3% (1/76) as very good, with 9.2% (7/76) missing answers. Distress at baseline was substantial, with a median DT score of 6 (IQR 4–7). Distress and general health showed weak correlation (Spearman’s ⍴=0.27, *p* = 0.025) at baseline, but not when including data of all visits and treating them as repeated measures (rmcorr r = -0.04, *p* = 0.604).

**Fig. 6:**
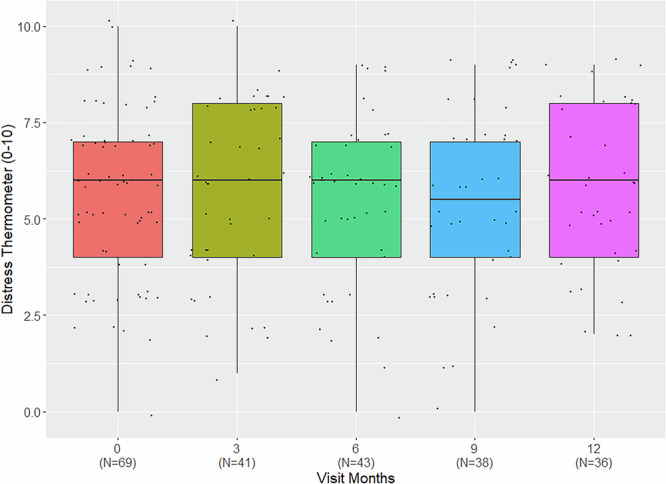
Distress over study course. Boxplot summarizing self-reported distress from 0 (no distress) to 10 (extreme distress) within the last week using the Distress Thermometer in kidney transplant recipients with impaired graft function for study visits (baseline-12 months).

Distress remained high throughout the study course (Fig. 6) and neither study intervention nor the visit number had significant effect on the level of distress reported (*p* = 0.787 and *p* = 0.871, respectively) in a LME model with patient as random effect, and treatment group and visit number as fixed effects.

Patients mostly reported emotional and physical problems as reasons for distress. Ten items were consistently reported in >30% of the surveys: exhaustion (61.9%), sleep (51.6%), pain (48.2%), concerns (46.2%), mobility (43.8%), dry/itching skin (35.5%), diarrhea (35.0%), digestive problems (32.3%), sadness (31.2%), and fears (30.3%).

### Validation of prediction model

**Fig. 7:**
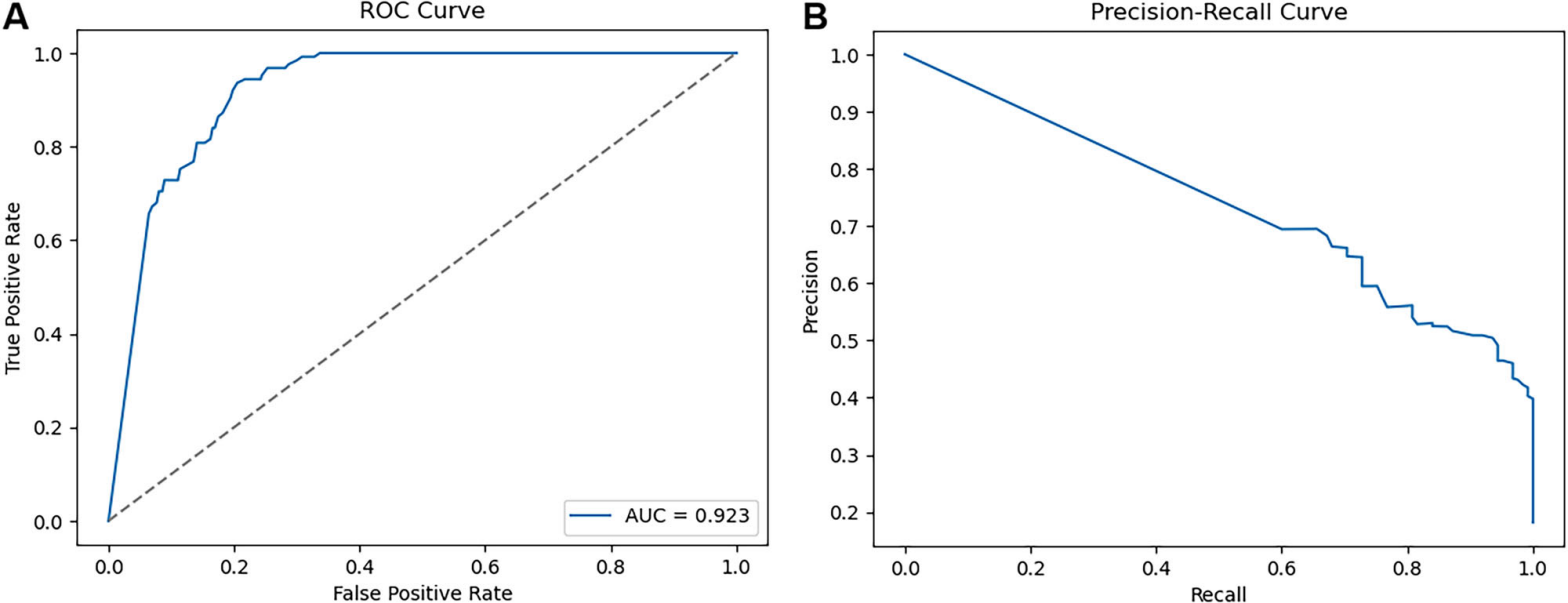
Model validation within study cohort. Model performance validation in *N* = 76 study cohort with **A** receiver operating characteristics (ROC) analysis, **B** precision-recall analysis.

We validated the risk prediction model prospectively in all 76 study patients, irrespective of study intervention. We used the risk score at study inclusion and evaluated whether the patients experienced graft loss within 360 days after inclusion (as used for training). The overall model performance was AUC-ROC (area under curve of the receiver operating characteristics): 0.923, AUPRC (area under the precision recall curve): 0.644 (Fig. 7). Based on the prespecified decision thresholds implemented as risk categories within the EHR integration, we found following predictive metrics: red area (high risk; threshold 0.7894)–sensitivity 0.840, specificity 0.834; yellow area (medium risk; threshold 0.5016)–sensitivity 1, specificity 0.604.

### Post-study user survey

A post-study user survey was performed to analyze whether underutilization of the risk score was a potential reason for the lack of efficacy observed and to determine potential barriers. The reported utilization differed highly between physicians (one physician reported 100% utilization, while all remaining physicians reported 0–30%; mean 29%, SD 37%), precluding meaningful stratified analysis by clinician-level uptake. Users described the following reasons as main barriers to use the system: lack of immediate clinical consequence or no perceived need for risk stratification, lack of time during the visit, no direct integration into workflow since the integration was provided in another tab of the EHR, no alerts in case of high-risk scores.

## Discussion

This randomized trial evaluated whether an EHR-embedded AI model estimating 1-year graft loss risk changes communication and SDM-related outcomes in post kidney transplant care. To our knowledge, PRIMA-AI is the first randomized study to test real-world implementation of an AI-based risk prediction tool in kidney transplantation, shifting the focus from predictive performance to patient-centered care processes. This perspective is relevant for digital health research because outcomes such as conversation frequency and SDM measures are rarely assessed in AI implementation studies, despite being plausible pathways through which risk prediction could improve patient-centered outcomes^1^.

The intervention showed no effect on the primary outcome or any secondary outcome. Conversation frequency about treatment options after graft loss was similar between groups, and no differences were observed in SDM-related measures (including CPS/CPS-post and CollaboRATE), physician-patient relationship ratings, distress, or clinical outcomes. These findings suggest that, in its current form and implementation, providing AI risk estimates in the EHR did not measurably change patient-perceived communication or decision-making processes over 12 months.

Several factors may explain the observed lack of efficacy. First, clinician uptake of the tool appears to have been low. Notably, only one physician reported high utilization, while all others reported using the tool in 0–30% of intervention visits, making it unlikely that the intervention reached sufficient exposure to influence outcomes. The post-study user survey indicated limited use of the AI risk information, with barriers including time constraints, perceived limited utility in routine visits, and suboptimal workflow integration (e.g., requiring navigation rather than appearing in-context or via alerts). Regarding the latter concern, the risk score was in fact directly implemented into the EHR and navigating towards the risk score was possible with one click and without loading time. Another form of implementation (e.g. into the documentation field) was not feasible. In the current EHR, other relevant information such as laboratory results, medical reports or transplant-specific information need to be accessed in the same way. Providing alerts would be a way to circumvent this problem but regarding the fact that several physicians deemed the risk score not relevant for their daily work, such implementation could also negatively affect the physicians’ workflow, which is why we opted against it in the development phase. It is obvious that an intervention that is rarely accessed is unlikely to change clinician behavior or patient experience.

Second, the rate of graft loss conversations in the control group was much higher than anticipated. The sample size calculation assumed conversation frequencies around 10%–15% based on prior reports^12,19^, whereas the observed control-group conversation frequency was approximately 40%, which reduces the study’s power. A plausible reason is the study’s eligibility criterion: by enrolling only kidney transplant recipients with impaired graft function -estimated glomerular filtration rate (eGFR) < 30 mL/min/1.73 m²–we selected a population in which clinicians may already initiate prognostic and planning discussions more often because urgency is higher. In that context, an additional AI “trigger” may have limited additional benefit unless it changes timing, personalization, or rigor of such conversations.

Third, the clinical relevance of conversations about kidney replacement therapy after graft loss may vary across individuals even within the same eGFR range. An eGFR threshold captures severity but not the trajectory. Some patients with eGFR <30 mL/min/1.73 m² may remain relatively stable, and detailed planning discussions may not be prioritized within a 12-month window. Physicians may even consider it psychologically harmful to discuss dialysis initiation with a stable patient too early. Therefore, it is not clear what the optimal proportion of patients is with whom such a conversation should be performed.

The numerically lower graft loss rate in the intervention group (3/36 [8%] vs 7/40 [18%]; *p* = 0.32) should be interpreted cautiously. With only 10 events, the confidence interval is wide and fully compatible with no difference. Moreover, baseline eGFR was numerically higher in the intervention group (27.1 vs 24.6 mL/min/1.73 m²), suggesting that control-group patients may have been closer to graft failure at enrollment. A causal effect of the intervention on graft survival is also implausible on mechanistic grounds: the risk model predicts short-term outcomes that are largely non-modifiable with currently available interventions, and clinician utilization of the tool was low, making it unlikely that it triggered treatment changes sufficient to prevent graft loss.

A further interpretive point arises from discordant response patterns across survey measures. A high proportion of participants of ~80% completed CPS-post at least once, whereas a smaller proportion of ~40% reported a conversation about treatment options after graft loss. This discrepancy could indicate that CPS-post was sometimes answered with reference to the last decision in the encounter that was unrelated to graft loss planning. Alternatively, conversations about future therapy may have occurred but were not captured by the primary outcome, for example, if they occurred between study visits, discussions were brief, or framed in a way patients did not recognize as being about treatment options after graft loss. Importantly, there was no difference in CPS-post response rate between groups, making it unlikely that survey behavior explains the null intervention effect.

Similarly, the absence of effects on CollaboRATE is not unexpected given the intervention’s scope. The CollaboRATE score was designed to assess SDM for the most recent decision in a visit, which may not concern graft loss planning. An intervention designed to influence whether a particular topic is addressed may not affect global SDM ratings unless the targeted topic is discussed and informs a salient decision for the patient at that encounter. Therefore, null CollaboRATE findings primarily suggest no measurable change in overall decision-making quality across routine visits rather than ruling out subtle topic-specific effects.

This study has limitations that affect inference. Recruitment ended before the planned sample size was reached (actual recruitment was 62% [76/122] of the planned sample size). Although the primary outcome estimates do not suggest a trend toward benefit, the smaller sample size reduces precision, particularly for secondary outcomes and subgroup analyses. Attrition was substantial (the final drop-out rate was 30.3% [23/76]) and resulted from both medical events (including graft failure and death) and nonmedical reasons (mostly consent withdrawal and in one case loss to follow-up). The protocol was amended to end follow-up at 12 months, with cancellation of the planned extended observation to 24 months because of funding constraints and high attrition, limiting evaluation of longer-term effects. Finally, the primary outcome was patient-reported and therefore subject to recall and interpretation; while this captures patient experience, it may miss brief or fragmented discussions.

Whether longer follow-up would have altered the findings deserves consideration. On one hand, some patients with eGFR <30 mL/min/1.73 m² remain relatively stable, and a clinically actionable change in risk trajectory may require more than 12 months to emerge. Additionally, since the risk model was activated at month 3 after randomization, the effective exposure period was approximately 9 months, and a longer observation could have allowed more encounters with evolving risk estimates. On the other hand, several factors argue against a meaningful benefit from extended follow-up. The primary outcome showed no trend toward a difference at 12 months, making it unlikely that additional time would have revealed an effect. Attrition was already substantial at 12 months, and further dropout would have reduced statistical power and introduced survivorship bias favoring stable patients in whom graft-loss discussions may be least relevant. Finally, clinician uptake of the tool was low and, based on post-study survey responses, unlikely to have increased over time.

An additional implication concerns intervention strategy. PRIMA-AI primarily relied on risk information as an indirect lever: higher perceived risk was expected to increase conversations, which would then improve SDM-related outcomes. The null findings suggest that this causal chain is fragile in routine care. Prediction did not reliably trigger conversations due to low uptake, and even when conversations occurred, their effect on downstream outcomes, such as timely access planning or patient satisfaction remains uncertain. More broadly, predictive accuracy alone does not confer clinical actionability: a risk prediction is most valuable when it changes a decision–whether initiating nephroprotective treatment, clarifying patient preferences, or planning the transition to dialysis–that in turn changes an outcome, be it clinical (such as timely dialysis access) or process-related (such as reduced decisional conflict or improved advance care planning). Future AI interventions may therefore need to target SDM more directly, for example, by integrating patient-facing decision aids, structured preference elicitation, or value clarification tools linked to the risk estimate; generating tailored question prompts for patients and clinicians; or providing visit-level SDM checklists that facilitate deliberation, not only initiation of a topic. Such approaches could also improve measurement fidelity by aligning intervention components with SDM-specific outcomes (e.g., decisional conflict, knowledge, values-concordance). Future implementations should not only ensure that risk information reaches clinicians but also define explicit, evidence-based care actions linked to specific risk thresholds.

Beyond targeting SDM processes directly, future implementations should address the workflow barriers identified in this trial. Concrete strategies include: (1) in-context display of the risk estimate within the documentation or visit summary screen rather than in a separate tab, reducing the navigation required; (2) threshold-based alerts that notify the clinician when a patient’s predicted risk crosses a predefined level or increases substantially between visits, thereby shifting from passive availability to active prompting; (3) integration into clinical order sets or care pathways, such that a high-risk classification automatically suggests referral to a vascular access consultation if needed or pre-dialysis education program; and (4) patient-facing components, for example a pre-visit summary or digital letter informing the patient that their kidney function trajectory warrants a conversation about future options, thereby creating a bilateral prompt. That complementary implementation components may be required to translate prediction into action is supported by the trial of Manz et al., who conducted a cluster-randomized trial in oncology investigating AI-based mortality prediction coupled with behavioural nudges to increase the rate of serious illness conversations (SIC). Their intervention combined machine-learning-based risk predictions with (1) weekly emails to clinicians comparing their SIC rates against peers’ rates, (2) weekly lists of high-risk patients, and (3) opt-out text messages to prompt SICs before encounters with high-risk patients^17,18^. This multi-component approach resulted in a higher rate of serious illness conversations and a lower rate of systemic therapy in the last 2 weeks before death, but had no effect on hospice length of stay or intensive care unit utilization^18^. Taken together, these findings underscore that the risk estimate itself may be necessary but not sufficient, and that future studies should test implementation components individually and in combination, for example, using a stepped-wedge or factorial design, to identify which elements drive uptake and downstream outcomes while remaining realistic for eventual routine deployment.

In summary, PRIMA-AI provides randomized evidence that passive availability of AI risk predictions within the EHR without strong workflow integration and with low clinician utilization did not increase conversation frequency or improve SDM-related outcomes in this cohort. Future work should prioritize implementation strategies that increase uptake (e.g., in-context display, alerting when risk increases, integration with actionable care pathways) and consider AI-enabled interventions that directly support SDM processes rather than influencing them only indirectly via conversation initiation.

## Methods

### Trial design and setting

PRIMA-AI is a prospective, randomized, 2-arm, parallel-group, single-center trial conducted in the outpatient kidney transplant center (KTC) of Charité–Universitätsmedizin Berlin, a tertiary care center in Germany performing ~200 kidney transplantations per year. Participants received routine post-kidney transplant follow-up (typically every 3 months) at the KTC, with clinical data documented in a proprietary EHR^19^. This report presents quantitative survey and clinical data from baseline through month 12 after randomization, including the primary outcome of the study. All subjects gave their written informed consent for inclusion before they participated in the study. The study was conducted in accordance with the Declaration of Helsinki, and the protocol was approved by the ethics committee of Charité–Universitätsmedizin Berlin (EA1/177/23 on August 08, 2023). The trial was preregistered at ClinicalTrials.gov (ClinicalTrials.gov number, NCT06056518, Date of registration September 21, 2023), and the protocol was published before^19^. Patient representatives were part of the scientific advisory board and were consulted during the design of the study.

### Eligibility criteria

Patients were eligible if they (1) provided written informed consent, (2) had a functioning kidney allograft, (3) were ≥12 months after transplantation, (4) had estimated glomerular filtration rate (eGFR) < 30 mL/min/1.73 m² according to CKD-EPI 2021 at the screening visit, (5) were aged ≥18 years, (6) were able to communicate in German, and (7) attended regular follow-up at the KTC^17^. Key exclusion criteria were multi-organ transplantation, eGFR >30 mL/min/1.73 m² at screening, <12 months after transplantation, age <18 years, inability to communicate in German, lack of regular follow-up at the KTC, and enrollment in another interventional study within the prior month^19^.

### Intervention and comparator

Comparator (usual care): Participants randomized to the control arm received routine post-transplant care according to local standard operating procedures and guideline-based management, including individualized visit frequency (commonly every 3 months in stable long-term follow-up) and standard management of immunosuppression, infection prophylaxis, and graft monitoring.

**Fig. 8:**
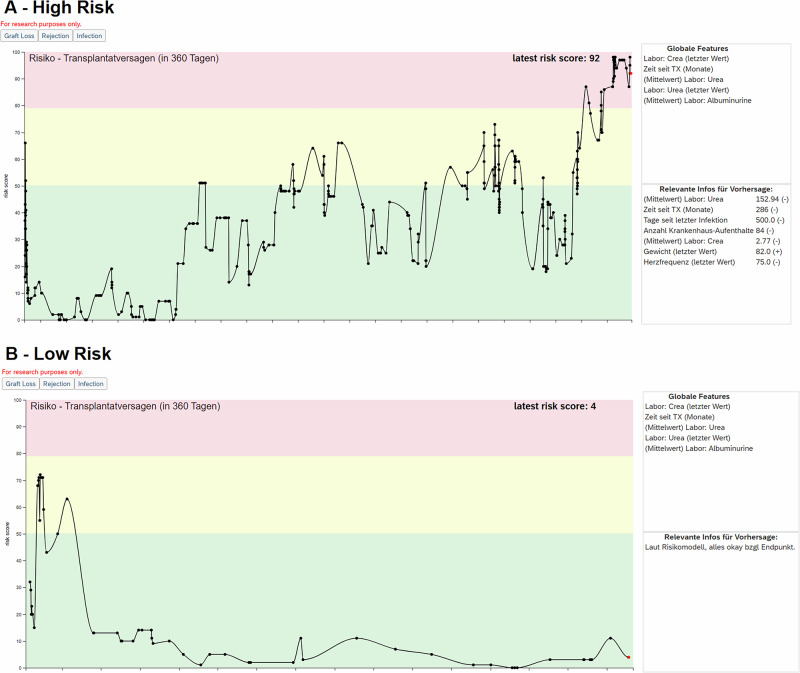
Sample screenshots of the risk score integration into the electronic health record. The screenshots show the risk trajectories over time for Graft Loss (“Transplantatversagen”) in **A** a high-risk patient, and **B** a low-risk patient. Time information was removed from the x-axis. The latest risk score is highlighted in red. Red (high risk), yellow (medium risk), and green (low-risk) areas are depicted to support the risk stratification. Relevant global and local features are provided as a basic explanation on the right. The risk-scores were precalculated on a daily basis. The authors are creators of the images and hold the copyright for the screenshot.

Intervention (AI-supported care): In addition to usual care, physicians treating patients in the intervention arm were provided with an EHR-integrated machine-learning decision-support system that estimated the individualized risk of graft loss within the subsequent year using recent clinical, laboratory, diagnosis, procedure, and histopathology data. The model provided a set of 5 global and local features for each risk score, and physicians were trained regarding the model outcome, training data used, model performance, and study protocol. The system was enabled beginning at month 3 after randomization. Physicians were free to use the risk estimates in consultations and also show them to patients^13^. If a high risk score was shown, the physicians were advised during the training to consider informing the patient about the risk and discussing potential need for return to dialysis or retransplantation, and planning aligned with patient preferences. Sample screenshots of the EHR integration for low-risk and high-risk patients are shown in Fig. 8.

### AI-based risk prediction model

The machine-learning model underlying the intervention was developed and internally validated using retrospective data from the same transplant center, as previously described^13^. In brief, the model uses gradient-boosted regression trees trained on structured clinical routine data from over 1500 kidney transplant recipients documented in the center’s EHR from 2000 until 2023. Each prediction is generated at the time of new data entry (e.g., laboratory assessment, new vital signs, new diagnosis entry) and estimates the probability of death-censored graft failure within the subsequent 360 days (90 days in the previously published version of the model). The model integrates approximately 300 features, including laboratory values (e.g., serum creatinine, eGFR), time since transplantation, number of prior transplantations, medication data, diagnostic codes, and derived variables such as gradients of successive laboratory measurements. The most influential global features are time since last transplantation, number of transplantations, serum creatinine, and eGFR. For each individual prediction, five local feature attributions are displayed alongside the risk score to provide case-level interpretability. Risk scores are categorized into low, medium, and high-risk using decision thresholds optimized on a development dataset. The model was retrained for the current study using the same algorithmic framework with a 360-day prediction window and prospectively validated within the trial cohort.

### Outcomes

Primary outcome: The primary outcome was the frequency (proportion) of patient-perceived conversations about treatment options after graft loss, compared between groups at 12 months after randomization.

Secondary outcomes: Secondary outcomes included (1) clinical outcomes (eg, proportion requiring dialysis therapy within follow-up, dialysis modality, dialysis access chosen, and emergency dialysis initiation) and (2) SDM-related and patient-reported outcomes. SDM-related measures included the CPS (adapted to the decision about kidney replacement therapy after graft loss), a post-decision CPS measure (CPS-post)^20^, and CollaboRATE (mean score and top score)^21,22^. Additional patient-reported measures included the Distress Thermometer (0–10), self-rated general health, and the Patient–Doctor Relationship Questionnaire (PDRQ-9)^23,24^. Surveys assessing the primary outcome and SDM-related measures were administered at baseline and at regular 3-month follow-up intervals through month 12. A post-study user survey was sent to all physicians treating at least 1 patient in the intervention group, consisting of questions about estimated utilization (in % of all intervention visits) of the AI system and reasons for non-utilization if applicable.

### Sample size

The planned sample size was 122 participants (61 per group), providing 80% power (*α* = 0.05) to detect an increase in conversation frequency from approximately 10%–15% in usual care to 40%–45% with AI-supported care, allowing for an estimated 10% annual dropout^19^. Enrollment was stopped before the target sample size (*n* = 122) was achieved because recruitment was slower than anticipated and due to funding restrictions. Study surveys were pilot tested in 5 patients/support persons before implementation. The study was initially designed to include an additional 12-month observation period after assessment of the primary outcome until 24 months after randomization. Due to the end of funding and considerable attrition within 12 months after randomization, the study protocol was changed to terminate the study after 12 months.

### Randomization and blinding

Participants were randomized 1:1 to AI-supported care or usual care using a predefined variable block randomization scheme via a web-based randomization service after eligibility confirmation and assignment of an individual patient identifier^25^. Personnel who enrolled and assigned participants to the interventions had no access to the random allocation sequence. The time of randomization was recorded. Blinding of participants and study staff was not feasible because of the nature of the EHR-embedded intervention.

### Statistical methods

The primary outcome (conversation frequency at 12 months) was compared between groups using Chi-square test, secondary medical outcomes were compared between groups either using Chi-square or Fisher’s exact test for small groups. Odds ratios were calculated using Fisher’s exact test. Descriptive statistics were reported as absolute and relative frequencies, mean ± SD for approximately normally distributed variables, and median (IQR) otherwise. To assess the association between preferred and experienced decision roles, repeated-measures correlation was calculated using the 5-point CPS, treating repeated surveys per participant as repeated measures (R package rmcorr)^26^. Mean differences between preferred and experienced roles were computed per participant (excluding missing values) and then summarized across participants as mean ± SD. For repeated measures outcomes (e.g., CollaboRATE and related items), LME models were fitted with participants and physicians as random effects and visit and treatment group as fixed effects. No imputation was performed. The number of patients included in each analysis varies by outcome and time point, because patients who withdrew or experienced medical events (e.g., graft loss, death) contributed questionnaire data up to their last completed visit. Denominators are reported with each analysis. Analyses were conducted in R version 4.5.1^27^.

### Use of large language models

Generative AI was used for linguistic refinements to improve clarity and readability of the manuscript and for pre-submission formatting.

## Supplementary information

Supplementary information

## Data Availability

Data availability Deidentified, individual participant data that underlie the results reported in this article, a data dictionary and the statistical analysis code will be shared immediately following publication. Data will be available indefinitely at 10.5281/zenodo.17916527. Information about treating physicians as well as potentially identifying items from the surveys will be excluded from the dataset for privacy reasons.

## Code availability

The underlying code for this study is not publicly available but may be made available to qualified researchers on reasonable request from the corresponding author.
